# Specific FRET Probes Sensitive to Chitosan-Based Polymeric Micelles Formation, Drug-Loading, and Fine Structural Features

**DOI:** 10.3390/polym16060739

**Published:** 2024-03-08

**Authors:** Igor D. Zlotnikov, Ivan V. Savchenko, Elena V. Kudryashova

**Affiliations:** Faculty of Chemistry, Lomonosov Moscow State University, Leninskie Gory, 1/3, 119991 Moscow, Russia; zlotnikovid@my.msu.ru (I.D.Z.);

**Keywords:** FRET probes, rhodamine 6G, chitosan, polymeric micelles, surfactants, stimulus-sensitivity, tumor microenvironment

## Abstract

Förster resonance energy transfer (FRET) probes are a promising tool for studying numerous biochemical processes. In this paper, we show the application of the FRET phenomenon to observe the micelle formation from surfactants, micelles self-assembling from chitosan grafted with fatty acid (oleic—OA, or lipoic—LA), cross-linking of SH groups in the micelle’s core, and inclusion and release of the model drug cargo from the micelles. Using the carbodiimide approach, amphiphilic chitosan-based polymers with (1) SH groups, (2) crosslinked with S-S between polymer chains, and (3) without SH and S-S groups were synthesized, followed by characterization by FTIR and NMR spectroscopy. Two pairs of fluorophores were investigated: 4-methylumbelliferon-trimethylammoniocinnamate—rhodamine (MUTMAC–R6G) and fluorescein isothiocyanate—rhodamine (FITC–R6G). While FITC–R6G has been described before as an FRET-producing pair, for MUTMAC–R6G, this has not been described. R6G, in addition to being an acceptor fluorophore, also serves as a model cytostatic drug in drug-release experiments. As one could expect, in aqueous solution, FRET effect was poor, but when exposed to the micelles, both MUTMAC–R6G and FITC–R6G yielded a pronounced FRET effect. Most likely, the formation of micelles is accompanied by the forced convergence of fluorophores in the hydrophobic micelle core by a donor-to-acceptor distance (**r**) significantly closer than in the aqueous buffer solution, which was reflected in the increase in the FRET efficiency (**E**). Therefore, **r(E)** could be used as analytical signal of the micelle formation, including critical micelle concentration (CMC) and critical pre-micelle concentration (CPMC), yielding values in good agreement with the literature for similar systems. We found that the **r**-function provides analytically valuable information about the nature and mechanism of micelle formation. S-S crosslinking between polymer chains makes the micelle more compact and stable in the normal physiological conditions, but loosens in the glutathione-rich tumor microenvironment, which is considered as an efficient approach in targeted drug delivery. Indeed, we found that R6G, as a model cytostatic agent, is released from micelles with initial rate of 5%/h in a normal tissue microenvironment, but in a tumor microenvironment model (10 mM glutathione), the release of R6G from S-S stitched polymeric micelles increased up to 24%/h. Drug-loading capacity differed substantially: from 75–80% for nonstitched polymeric micelles to ~90% for S-S stitched micelles. Therefore, appropriate FRET probes can provide comprehensive information about the micellar system, thus helping to fine-tune the drug delivery system.

## 1. Introduction

In the last decade, Förster resonance energy transfer (FRET) between fluorophore molecules [1,2,3,4] has been actively developed as a quantitative approach to determine a number of biochemical parameters in real time [5]. This approach turned out to be advantageous, providing high sensitivity and selectivity, since the FRET effect reflects the specific molecular organization in the system. In addition, changes in the FRET signal can be monitored online upon the supramolecular assembly self-organization process [6], which is undoubtedly an advantage over other methods such as electron microscopy, radioactive tagging, and dynamic light scattering. It is worth noting that the FRET signal can be used to create powerful sensors in the biological research and medical applications.

FRET can be observed when the emission spectrum of the donor overlaps with the excitation spectrum of the acceptor, and the distance at which the energy transfer can occur is limited to ~10 nm. The quantum yield of this energy-transfer transition, FRET efficiency (E), is determined by the donor-to-acceptor distance r [7,8,9,10]:

E = 1/(1 + (r/R_0_)^6^), (1)

where R_0_ is the Förster distance of the given pair donor–acceptor, which can range from 10 to 100 Å. The Förster radius R_0_ for fluorescein isothiocyanate (FITC) and rhodamine 6G (R6G) is ~50 Å, and for—4-methylumbelliferyl p-trimethylammoniocinnamate chloride (MUTMAC) and R6G is about 60 Å.

The choice of fluorophores for FRET probes is also justified by their potential as potential medicines: Rhodamine 6G and its derivatives [11,12], as well as Coumarin and derivatives, are model cytostatics proposed for use as medicines [13] and as model fluorophores for studying the loading degree.

Since even small changes in the donor–acceptor distance (r/R_0_) crucially affect FRET efficiency, the FRET-based approach can be considered as a powerful tool for the studies involving accurate estimation of the inter- and intramolecular distances, in the molecular dynamics assays, molecular interactions, and binding events. Interestingly, the FRET approach seems promising in the study of the formation and functional properties of polymeric micelles [14,15], the most popular drug delivery systems. Among advantages of polymeric micelles as drug carriers are their ability to encapsulate a wide array of hydrophobic and poorly soluble therapeutic agents, coupled with their propensity for prolonged circulation and passive tumor targeting through the enhanced permeability and retention (EPR) effect [6].

In this paper, we investigated the role of the micelles formation in FRET phenomenon, where the FRET effect is expected to be increased due to concentration and convergence of the donor–acceptor agents caused by its specific hydrophobic–hydrophilic phase distribution in micelles. Therefore, two main applications of FRET probes are considered: (1) Determination of the CMC and CPMC (critical micelle and pre-micelle concentrations, respectively) values, and (2) the study of the kinetics of the formation and destruction of S-S bonds in the tumor microenvironment using the example of stimulus-sensitive micelles from molecules of chitosan grafted with lipoic acid residues.

In the case of classical micelles, the spontaneous formation of spherical particles associated with surfactant molecules (SDS, Triton X-100, etc.) leads to the loading of the aromatic fluorophore molecules into the hydrophobic micelle core, which can be used in the observation of FRET during micelle formation [16]. The FRET phenomenon depends on the environment of the fluorophores (buffer, cationic/anionic/zwitterionic, or neutral surfactant): energy transfer on rhodamine is active in anionic/nonionic media [16].

We used surfactants (control systems with parameters (CMC) described in the literature) for validation of the FRET-based approach in order to proceed further in the investigation of grafted chitosan polymeric micelles. Recently, we suggested the approach where FRET was used as an effective tool for monitoring the formation of micro-/nano-gels [5,17]. We showed that the formation of chitosan nanogels promotes the interaction of pyrene covalently attached to chitosan with added model drug molecules of tryptophan (biologically active substance), which is necessary for the appearance of the FRET effect and which is not observed in the solution before nanogel formation.

The study of micelle formation is important from the point of view of creating smart delivery systems for antibacterial and antitumor drugs [18,19,20,21,22,23,24,25,26,27,28,29,30,31]. FRET is applicable for studying the formation of various types of nanoparticles based on polymers (chitosan, chitosan-PEG) and proteins (ovalbumin, casein, etc.) [32]. Nanoparticles, along with micelles, deserve special attention as promising drug carriers [33,34,35,36]. Polymeric micelles are promising carriers of a wide range of drugs, since they have a number of properties [37,38,39,40,41,42,43]: (1) the external hydrophilic shell ensures the colloidal stability of the system; (2) the internal hydrophobic core is necessary for the solubilization of drugs, which are often poorly soluble (which limits their use in medicine); (3) thermodynamic stability; (4) the possibility of obtaining biocompatible micellar structures; (5) increased permeability of the drug to target cells due to fatty acids; (6) wide possibilities for creating stimulus-sensitive delivery systems, for example, in tumors. For the latter, chitosan demonstrated pH sensitivity to a slightly acidic environment (tumors), and lipoic acid residues with S-S bonds between various polymer chains provided glutathione sensitivity [44]. Here, we propose to study the mechanisms of formation of such micelles using the FRET probe technique based on changes in the FRET efficiency and the distance between fluorophores during aggregation and disaggregation of amphiphilic molecules.

## 2. Materials and Methods

### 2.1. Reagents

Surfactants SDS (sodium dodecyl sulfate), Triton X-100 and zephirol (N-benzoyl-N,N-dimethyldodecan-1-ammonium chloride) were purchased from Reachim (Moscow, Russia). The fluorophores rhodamine 6G (R6G), fluorescein isothiocyanate (FITC), 4-methylumbelliferyl p-trimethylammoniocinnamate chloride (MUTMAC), 4-methylumbelliferone (MUmb); chitosan oligosaccharide lactate 5 kDa (Chit5), lipoic acid (LA), 1-ethyl-3-(3-dimethylaminopropyl) carbodiimide (EDC), N-hydroxysuccinimide (NHS), 1 M 2,4,6-trinitrobenzenesulfonic acid, and the enzyme α-chymotripsin from bovine pancreas (EC 3.4.21.1, ≥40 units/mg protein) were purchased from Sigma-Aldrich (St. Louis, MO, USA).

### 2.2. Synthesis of Chitosan Grafted with Lipoic Acid (Chit5-LA) and Oleic Acid (Chit5-OA)—Micelles Preparation

The synthesis of modified chitosan was carried out as described by us earlier with some modifications [45,46,47]. Chitosan was dissolved in 1 mM HCl solution (10 mg/mL) and then the pH was adjusted to 7.4 using 0.1 M phosphate buffer. Lipoic acid was dissolved in PBS/EtOH (50/50 *v*/*v*) to a concentration of 20 mg/mL. NHS and EDC were dissolved in EtOH (50 mg/mL). The crosslinking reaction was carried out using a carbodiimide approach, for which the solutions described above were mixed so as to obtain the Chit5/LA/EDC/NHS mass ratios = 1/0.33/3/1, for OA 1/0.35/3/1. The mixture was incubated for 6 h at a temperature of 50 °C. The product was then purified by three-stage dialysis against water (12 h × 3, cut-off 3.5 kDa). The polymer was freeze-dried at –70 °C.

Amphiphilic chitosan-based polymers (1 nM–50 µM) were mixed with FRET probes (1 µM) in PBS (0.01 M, pH 7.4), and the mixtures were then incubated at 37 °C for 1 h. Micelle samples were obtained by ultrasonic treatment of solutions (22 kHz) for 15 min with constant cooling in an ultrasonic device (Cole-Parmer, Vernon Hills, IL, USA). Micellar solutions were extruded (5-fold, 400 nm membrane, Avanti Polar Lipids). The free fluorophores were then separated by dialysis against PBS (with a cut-off mass of 8 kDa), and the degree of loading was then determined by fluorescence intensity: (1) For MUTMAC λ_exci_ = 360 nm, λ_emi_ = 450 nm; (2) for R6G λ_exci_ = 515 nm, λ_emi_ = 550 nm; (3) for FITC λ_exci_ = 490 nm, λ_emi_ = 520 nm were used.

### 2.3. Characterization of Chitosan Grafted with Lipoic Acid (Chit5-LA)

The characterization of chitosan grafted with lipoic acid (Chit5-LA) was carried out by the methods of FTIR, ^1^H NMR spectroscopy, atomic force microscopy, and circular dichroism spectroscopy.

FTIR spectra of Chit5, LA, OA, Chit5-LA, and Chit5-OA were recorded using an FTIR microscope MICRAN-3 and Bruker Tensor 27 spectrometer equipped with a liquid-nitrogen-cooled MCT (mercury cadmium telluride) detector, as described earlier [45,48].

^1^H NMR spectra of samples (7–10 mg/mL in D_2_O) were recorded on a Bruker Avance 400 spectrometer (Germany, 400 MHz). FTIR and NMR spectroscopy was used to calculate the modification degree of chitosan.

Circular dichroism spectroscopy (Jasco J-815 CD Spectrometer, Tokyo, Japan) were used to estimate the deacetylation degree in Chitosan, which amounted to (92 ± 3)%.

Atomic force microscopy (AFM microscope NTEGRA II) was used to visualize polymeric micelles based on grafted chitosan and compare it in terms of shape and size with nonmodified chitosan.

The degree of chitosan modification by fatty acid residues was determined by a well-proven method of spectrophotometric titration of amino groups using 2,4,6-trinitrobenzenesulfonic acid forming colored adduct with amino groups (absorption at 420 nm). To 300 µL of solutions of modified and unmodified chitosan (0.03–0.2 mg/mL) in 0.02 M Na-borate buffer (pH 9.2), 3 µL of 1 M solution of trinitrobenzenesulfonic acid (TNBS) was added, and then kinetic curves at 420 nm (A420) were recorded for an hour. The grafting degree was calculated from the change in A420 relative to unmodified chitosan.

Hemolytic activity and thrombogenicity are the primary parameters for evaluating the safety of medical formulations. For chitosan and polymer micelles in concentrations up to 1 mg/mL, the values of hemolytic activity and thrombogenicity did not exceed 1–2%.

### 2.4. FRET Probes for Determination of CMC for Micelles Formed from Surfactants and Chit5-LA

#### 2.4.1. Determination of CMC for Micelles Formed from Surfactants

FRET probes are two pairs of fluorophores FITC–R6G and MUTMAC–R6G, where for both, R6G is the acceptor. We chose surfactants zephirol, Triton X-100, and SDS as amphiphilic compounds for studying micelles formation.

The excitation and emission spectra of fluorescence were recorded on the device Varian Cary Eclipse fluorescence spectrometer (Agilent Technologies, Santa Clara, CA, USA). For FRET probe 1 (MUTMAC + R6G), λ_exci_ = 360 nm, λ_emi_ = 450 nm (donor), and 550 nm (acceptor) were used. For FRET probe 2 (FITC + R6G), λ_exci_ = 460 nm, λ_emi_ = 520 nm (donor), and 550 nm (acceptor) were used.

The final concentration of fluorophores was 1 µg/mL. Fluorophore emission and excitation spectra were recorded for each separately and for a donor–acceptor mixture in a buffer solution (PBS 0.01 M, pH 7.4) in the absence of surfactants and in its presence of various amounts.

FRET efficiency E was calculated as

E = 1 − F_DA_/F_D_ (2)

for MUTMAC + R6G pair and as

E = F_AD_/F_A_ − 1 (3)

for FITC + R6G pair. Where F_DA_ and F_D_—the intensities of donor fluorescence in the presence and absence of the acceptor, respectively; F_AD_ and F_A_—the intensities of acceptor fluorescence in the presence and absence of the donor, respectively.

The ratio r/R_0_ was calculated as an analytical signal of micelle formation;

r/R_0_ = (1/E − 1)^(1/6) (4)

where r is the distance between donor and acceptor and R_0_ is Förster radius. Förster distance was calculated based on an assumption that orientation factor (κ^2^) is 0.667. Critical micelle concentration (CMC and CPMC) was estimated using x-coordinate of a point on the right branch of the graph (r/R_0_ versus surfactant concentration) with the value r/R_0_ equal to the initial one (for a pair of fluorophores in a buffer solution without surfactants).

#### 2.4.2. Determination of CMC for Polymeric Micelles

We chose chitosan grafted with lipoic acid (Chit5-LA) that formed S-S bonds, and as a control, chitosan grafted with oleic acid (Chit5-OA), as amphiphilic compounds for studying micelles formation.

The formation of S-S bonds between Chit5-LA polymeric chains was studied using FRET probes, registering their fluorescence as described above. First, dithiothreitol was added to the self-assembled Chit5-LA samples (0.05 mg/mL) to the final concentration of 0.2 mg/mL, and incubated for 30 min at 37 °C, followed by oxidized glutathione GSSG addition to the final concentration of 2–5 mg/mL. The fluorescence values were recorded before and after the addition of each component.

#### 2.4.3. Flow Cytometry for Micelle Formation Study

A CytoFLEX S flow cytometer (Beckman Coulter) was used to study micelles with fluorophore (R6G). The polymers (Chit5-LA, Chit5-(oleic acid)) were incubated with pure rhodamine 6G (5 µg/mL) for 15 min, then treated with ultrasound. We used a 488 nm laser for excitation. The fluorescence emissions were collected using a 585/42 nm bandpass filter for 30,000 micelles for each sample. The collected data were then analyzed using CytExpert software (v. 2.0).

#### 2.4.4. Release of R6G from Micelles by Addition of Reduced Glutathione as Thiol-Disulfide Exchange Agent (Tumor Microenvironment Model)

R6G-loaded micelles formed from self-assembled polymers (Chit5-LA and control Chit5-(oleic acid) without S-H bonds) were prepared in PBS (pH = 7.4, 0.01 M) after ultrasound treatment of 1 mL of each sample: polymer solution (2 mg/mL) + R6G solution (0.1 mg/mL). Further, reduced glutathione (as thiol-disulfide exchange agent) was added to the samples to destroy S-S bonds in micelles at concentrations of 0, 0.2, and 3 mg/mL. Release of R6G from micelles was studied using dialysis technique (6–8 kDa cut-off, 150 rpm) to external 10 mL PBS buffer solution at 37 °C. R6G in external solution was detected by absorption at 515 nm and fluorescence intensity at λ_exci_ = 515, λ_emi_ = 550 nm.

### 2.5. Enzyme Activity Studies for Determination of the Fluorophore Inclusion Degree in Micelles

The catalytic activity of α–chymotrypsin was determined fluorometrically on the device Varian Cary Eclipse fluorescence spectrometer (Agilent Technologies, Santa Clara, CA, USA). The reaction rate was measured at λ_exci_ = 360 nm, λ_emi_ = 450 nm, and T = 37 °C in PBS (0.01 M, pH 7.4) by the accumulation of the fluorescent product (MUTMAC --> MUmb): specific parameters are indicated in the captions of the tables and figures. The concentration of chymotrypsin was optimized as follows: We varied the concentration of chymotrypsin in the range of 0.05–3 µM, and chose the optimal concentration of 0.4 µM so that the initial section of the kinetic curve was linear for at least 1–2 min and the substrate was consumed within about 2–5 min, and not instantly. This approach allowed the determination of the concentrations of the MUTMAC fluorophore substrate from 0.01 mM to 1 mM.

## 3. Results

### 3.1. Article Design

**Figure 1:**
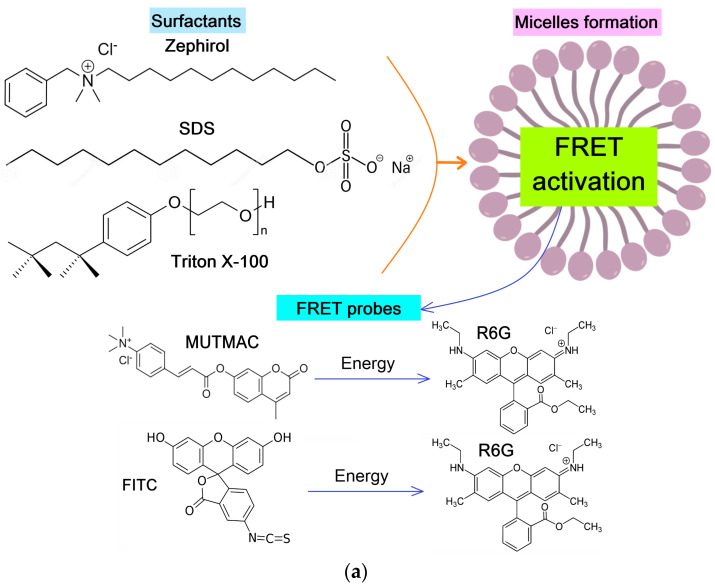

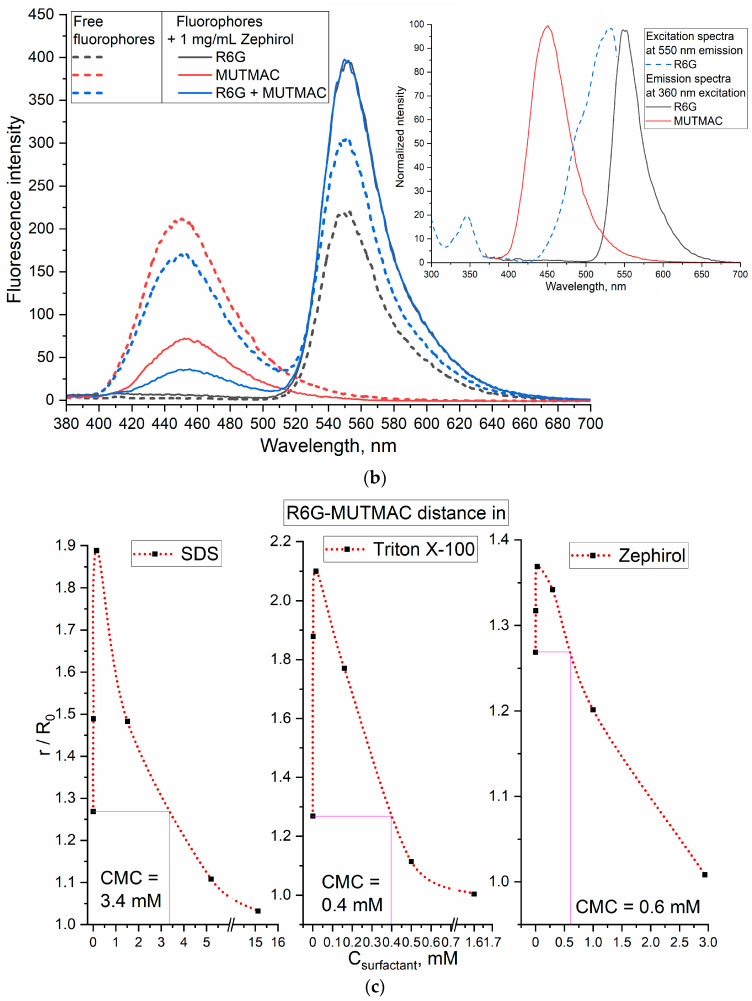

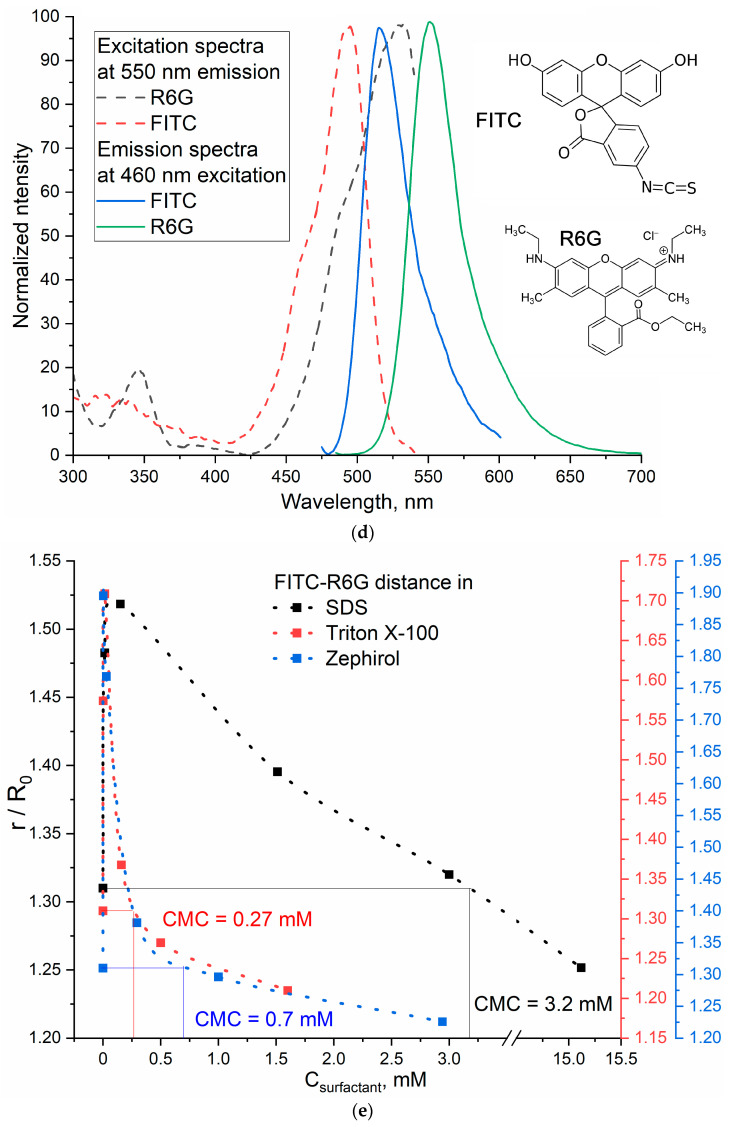
(**a**) Experiment design: FRET as an indicator of micelle formation from surfactants. (**b**) Emission fluorescence spectra of MUTMAC, R6G alone, and its mixtures 1 to 1 (1 µM/1 µM) in PBS buffer solution (0.01 M, pH 7.4) and in the presence of 1 mg/mL of the surfactant zephirol. The excitation wavelength is 360 nm. The insert shows the excitation and emission spectra of these fluorophores in PBS. (**c**) The dependences of r/R_0_ (MUTMAC–R6G) on the surfactants’ concentration; r—the distance between donor and acceptor, and R_0_ is Förster radius. (**d**) The excitation and emission spectra of FITC and R6G fluorophores in PBS at excitation wavelength 460 nm. (**e**) The dependence of r/R_0_ (MUTMAC–R6G) on the surfactants’ concentration (r is the distance between donor and acceptor and R_0_ is Förster radius). T = 22 °C.

The present work is aimed at studying the applications of the FRET effect as a selective indicator of surfactant molecules aggregation and as a tool for studying the promising drug carriers—polymeric micelles formed from chitosan-fatty acid conjugates. The first stage of the work is the validation of the FRET probe technique with classical surfactant micelles: we study the effect of charge, size, geometry, and degree of surfactants aggregation in micelles on the FRET effectiveness, and its correlations with micelle formation (CMC, CPMC). On the basis of this, the developed FRET technique, using chosen donor–acceptor pairs, was used to study the mechanisms of formation of stimulus-sensitive polymer micelles (with S-S bonds) to the tumor microenvironment (low pH and increased concentrations of glutathione, GSH). We considered the formation of micelles from surfactants of different structure (cationic Zephirol, anionic SDS, and neutral Triton X-100) and modified polymers (chitosan grafted with fatty acid), where FRET occurs between two fluorophores pairs (R6G with MUTMAC or FITC) due to their convergence in the core of the micelle. Objects of research (Figure 1): (i) Two pairs of fluorophores, FITC–R6G and MUTMAC–R6G; (ii) surfactants zephirol, Triton X-100, and SDS; (iii) chitosan grafted with lipoic (with S-S bond forming function) and oleic (without S-S bond) acid.

### 3.2. FRET as an Indicator of Micelle Formation in Surfactants Solution

As pairs of fluorophores with the FRET function, we chose MUTMAC–R6G and FITC–R6G (Figure 1a). The first pair is appropriate in terms of the ratio of the fluorescence intensities of the donor and acceptor (approximately 1 to 1), as well as the visual separation of emission peaks. The second pair: visually, the fluorescence peaks are not well resolved into components due to the close location of the bands of donor emission and acceptor absorption; however, this determines the high efficiency of FRET (E value Equation (1)). Such variability (spatial resolution)/(FRET efficiency) was studied here to select the optimal pair of fluorophores with the FRET function.

#### 3.2.1. MUTMAC–R6G Pair

Figure 1b shows the excitation and emission fluorescence spectra of MUTMAC (donor) and R6G (acceptor). The main components are the fluorescence peak of the donor at 450 nm and the acceptor—at 550 nm. The excitation wavelength was 360 nm, such that both MUTMAC and partially R6G would be excited, which makes it possible to monitor the fluorescence of both fluorophores.

The degree of fluorophore loading was controlled by changing the fluorescence intensity from the concentration of the added surfactant (Figure S1). For MUTMAC, an increase in emission intensity was observed during the formation of pre-micelles, and during the formation of micelles, fluorescence quenching occurred—a marker of the loading degree. At a concentration of surfactants of the order of 1 mg/mL, the degree of MUTMAC loading is 75–80%. In the case of rhodamine 6G, fluorescence ignition is mainly observed during the formation of pre-micelles and slight quenching during the formation of micelles based on Triton X-100 and Zephirol, and quenching during the formation of micelles of anionic SDS. The degree of loading of R6G at a concentration of surfactants of the order of 1 mg/mL can be estimated as 80–85%. The fluorescence emission spectra of R6G in free and micellar form are shown in Figure S1e. A shift of the maximum position to 5–10 nm is observed due to the inclusion of fluorophores in the micelles hydrophobic areas.

**Figure 2:**
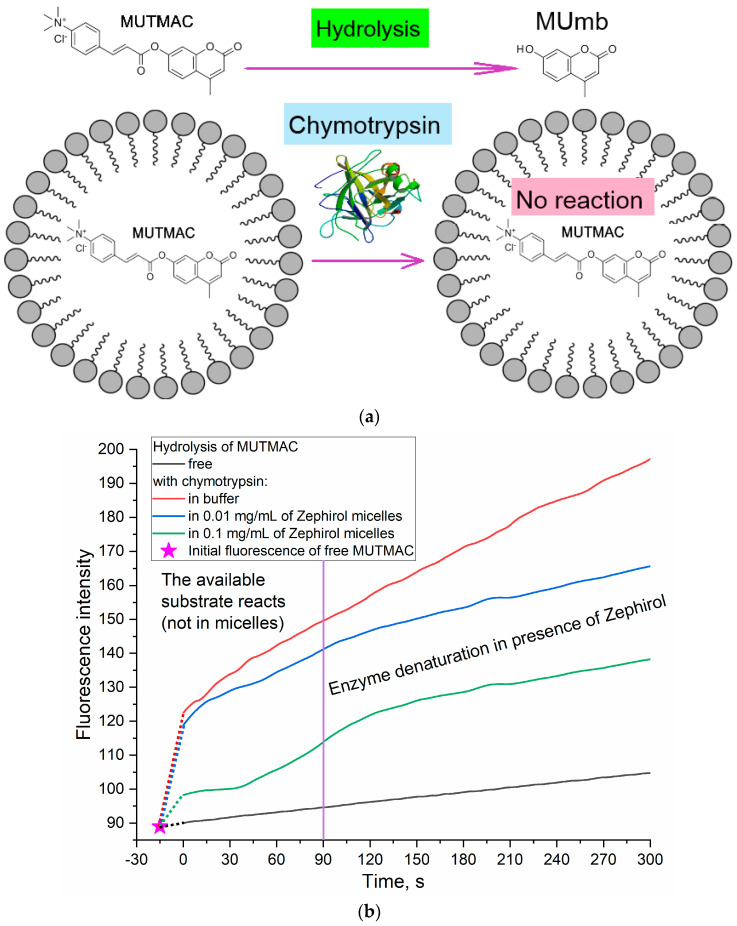
(**a**) Experiment design: determination of the fluorophore inclusion degree in micelles by enzymatic activity. (**b**) Kinetic curves of MUTMAC (0.1 mM) hydrolysis in the presence/absence of chymotrypsin (0.4 µM) and various concentrations of zephirol. λ_exci_ = 360 nm, λ_emi_ = 450 nm. PBS (0.01 M, pH 7.4). T = 37 °C. The reaction rate was determined by the initial spike in the fluorescence intensity of the product, and not by the tangent of the tilt angle, since the enzyme is partially denatured. The purple vertical line indicates >10% denaturation of the enzyme.

To quantify the formation of micelles from surfactants, it is necessary to select the target signal: the most pronounced is FRET efficiency (E value—Equations (1)–(3)) and the ratio r/R_0_ (Equation (4)), characterizing the distance between two molecules of the fluorophore: the donor and acceptor. r/R_0_ is directly related to the formation/disaggregation of micelles: (1) The addition of small amounts of surfactant to the system leads to the increasing the donor–acceptor molecules distance (Figure 1c); (2) The formation of micellar structures is reflected in the convergence of fluorophores due to its incorporation into the hydrophobic core of micelles, enhancing with the increase in surfactant concentrations. Therefore, the dependences of r/R_0_ on surfactants’ concentrations has a maximum, which means the initial process of the surfactant molecule aggregation (pre-micelles). The critical pre-micelle concentration (CPMC) can be determined from the position of the maximum curve. However, another analytically significant parameter is the critical micelle concentration (CMC). In this case, the CMC corresponds to a point on the right branch of the graph with the value r/R_0_ equal to the initial one (for a pair of fluorophores in a buffer solution without surfactants)—Figure 1c.

#### 3.2.2. FITC–R6G Pair

Figure 1d shows the excitation and emission fluorescence spectra of FITC (donor) and R6G (acceptor) separately from each other in a buffer solution. The main components are the fluorescence maximum for the donor observed at 520 nm and at 550 nm for the acceptor. The excitation wavelength was 460 nm for the selective observation of the FITC emission peak.

The degree of FITC and R6G loading was controlled by changing the fluorescence intensity from the concentration of the added surfactant (Figure S1d). For R6G, the observations are described above. In the case of FITC, an interesting fact is observed: the dependence of the fluorescence intensity on the concentration of surfactant is a curve with a minimum corresponding to the formation of pre-micelles and a right shoulder corresponding to the compactization of surfactant molecules into micelles.

In this system, it is most informative to determine the r/R_0_ ratio by the igniting of the acceptor (R6G) fluorescence intensity. Similarly to the MUTMAC–R6G pair considered above, the dependences of r/R_0_ on C_surfactant_ with a maximum are obtained for the FITC–R6G pair. Graphically, the points corresponding to CMC are marked in Figure 1e. The MUTMAC–R6G pair is more sensitive than FITC–R6G to the formation of micelles from charged surfactants, since the value of r/R_0_ changed significantly, and, in addition, the visual separation of the peaks of fluorescence emission makes it possible to estimate the values of CMC, CPMC, etc., much more accurately.

#### 3.2.3. Comparison of CMC Values Obtained Using Two FRET Probes and the Literature Data

Based on the plots given in Figure 1b,d (distances between the fluorophore donor and acceptor plotted on the concentration of surfactants), the CMC values were graphically determined (the results are presented in Table 1). The data obtained using two FRET probes coincide within the margin of error and satisfy the literature data obtained using fast titration method with ionic organic dyes. This means that the technique of FRET probes allows us to study the mechanisms of micelle formation and determine not only CMC, but also CPMC, which was previously available only by indirect methods.

*The effect of surfactants charge on FRET efficiency*. The largest maximum on the graph of r/R0 as a function of surfactant concentration (Figure 1c) is achieved for neutral Triton X-100 (2.1 units) and decreases for anionic SDS (1.9 units) and cationic Zephirol (1.37 units). This difference between anionic and cationic surfactants can be attributed to the positive charge on the FRET probe itself; therefore, in the case of «+»–charged surfactants, a convergence of fluorophores is observed due to repulsion from charged surfactant groups (Figure 1). By the magnitude of the maximum on the r/R_0_ curve, it is possible to judge the charge effect of surfactants on the micelles formation semi-quantitatively.

*The effect of micellar size on FRET efficiency*. Micellar size affects the distance between fluorophores, and it is therefore important to monitor the right branch of graphs of r/R_0_ versus surfactant concentration (Figure 1c). For classical surfactants, the curves exceed r/R_0_ ≈ 1, whereas for large chitosan polymer micelles (size 100–200 nm), the r/R_0_ is significantly larger than 1 (shown in Section 3.4.2). The highest CMC value is typical for surfactants with a low molecular weight—SDS, an order of magnitude lower CMC values are typical for surfactants with a high molecular weight such as Triton X-100 and Zephirol—due to multipoint interactions. The effect of the surfactants charge on the CMC is rather pronounced: the smallest CMC values are characteristic for uncharged surfactants. At the same time, Triton X-100 is characterized by a higher aggregation degree of 143 versus 50 for SDS [49,50]. This is reflected as an in increase in the sharpness of the peak r/R_0_ vs. C_surfactant_, which indicates the sensitivity of the presented FRET probes. Due to the charged groups in R6G and MUTMAC, these fluorophores can interact with anionic groups in surfactants, and specifically with the sulfogroup in SDS. This affects the observed FRET: the r/R_0_ parameter varies from 1.3 to 2.0 units; and in the case of cationic zephirol, r/R_0_ varies only slightly from 1.27 to 1.37 units. Thus, using the FRET technique, it is possible to judge the degree of aggregation and the size of micelles.

Additionally, we showed the *specificity of the probes to different types of micelles in terms of the FRET signal*. The **MUTMAC–R6G pair** is more specific than FITC–R6G to the formation of micelles from charged surfactants, due to the visual separation of fluorescence peaks. In addition, MUTMAC is a fluorescent substrate and can be used to study enzyme activity using the FRET phenomenon in the micellar systems or even in the living cells.

At the same time, the **FITC–R6G pair** is characterized by a higher degree of inclusion in the core of micelles >80–85% (for surfactant micelles at concentration higher than 1 mg/mL), and for the more hydrophilic MUTMAC, this value is about 75% (as can be judged from the fluorescence data). The difference in the inclusion degrees of fluorophores affects the sensitivity of the FRET probe and the spike in the analytical signal r/R_0_ (Figure 1).

**Table 1:** Critical micelle concentrations (CMCs) and critical pre-micelle concentrations (CPMCs) for surfactants determined using FRET probes in comparison with the literature data. PBS (0.01 M, pH 7.4). T = 22 °C.

Surfactant	CPMC, µM	CMC, mM		
		FRET Probe 1: MUTMAC + R6G	FRET Probe 2: FITC + R6G	Literature Data
SDS (sodium dodecyl sulfate)	15 ± 4	3.4 ± 0.1	3.2 ± 0.3	3.32 ± 0.01 [51]
Triton X-100	16 ± 3	0.39 ± 0.05	0.27 ± 0.08	0.3 ± 0.01 [51]
Zephirol (benzalkonium chloride)	4 ± 1	0.6 ± 0.1	0.7 ± 0.2	0.6 mM [52]

### 3.3. Determination of the Fluorophore Inclusion Degree in Micelles by Enzymatic Activity

A complementary approach to the FRET probes technique to determine the fluorophore loading degree in the micelles and the micelle formation (CMC) is the use of enzyme catalytic activity. α-Chymotrypsin (proteinase) catalyzes the hydrolysis reaction of MUTMAC to 4-methylumbelliferon (MUmb) (Figure 2) accompanied by the ignition of fluorescence at 450 nm (MUmb fluorescence). Upon formation of the micellar structures from Zephirol, MUTMAC enters the hydrophobic core; therefore, it becomes inaccessible for enzymatic reactions. Thus, with an increase in the surfactant concentration, there would be a decrease in the apparent reaction rate due to a decrease in the effective concentration of the substrate in aqueous phase. This experiment was specially designed so that the micelles were loaded with fluorophore, but not separated by dialysis, so that part of the fluorophore would not be in the micelles. This is to show the applicability of the method for determining fluorophore loading by enzymatic reactions based on the amount of fluorophore remaining outside the micellar structure.

According to the values of the fluorescence intensity changes at 450 nm (initial splash, which corresponds to the release MUmb product), and in comparison, with an aqueous solution, it is possible to judge the amount of fluorophore loaded into the micelle core: 20% of the fluorophore was screened by the surfactant at C_zephirol_ 0.01 mg/mL, and 65% was loaded in the micelles at C_zephirol_ = 0.1 mg/mL. The estimated CMC value calculated using the enzyme technique is 0.25 mM (=0.1 mg/mL), close to those given in Table 1 obtained using FRET probes approach (R6G with MUTMAC and FITC). Given that the surfactant can cause denaturation of the enzyme after 1–2 min (for chymotripsin) (Figure 2), the initial reaction rate should be used as a relevant analytical signal. The results obtained using enzymatic techniques for loading fluorophores into micelles are in good agreement with those obtained using FRET probes (section above). However, using chymotrypsin, it is possible to more accurately determine the distribution of fluorophores in the micellar system.

### 3.4. Formation of Polymeric Micelles as Assessed by FRET Probes

#### 3.4.1. Self-Assembled Amphiphilic Chitosan Grafted with Lipoic and Oleic Acid Residues

The first part of the work was devoted to the validation and optimization of the FRET technique for studying micelle formation. During the validation of the technique, a more sensitive MUTMAC–R6G FRET pair was selected. The main practical interest is, rather, polymeric micelles that are widely used for drug delivery. Accordingly, with regard to self-assembled amphiphilic chitosan grafted with lipoic and oleic acid residues, we aimed to study the mechanism of formation of the micelles, as well as to study the subtle nature of stimulus sensitivity due to loosening of the 3D structure of chitosan in a weakly acidic medium and the reducing of S-S bonds to S-H in the presence of glutathione as a model tumor microenvironment [53,54,55].

**Figure 3:**
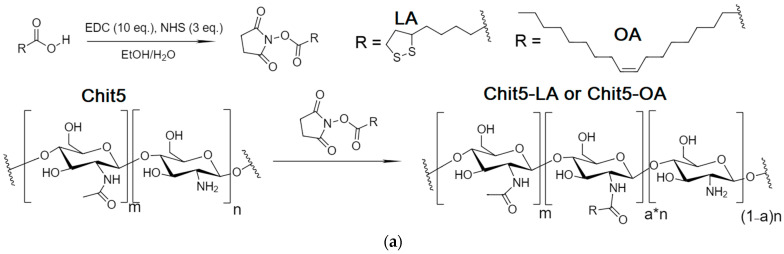

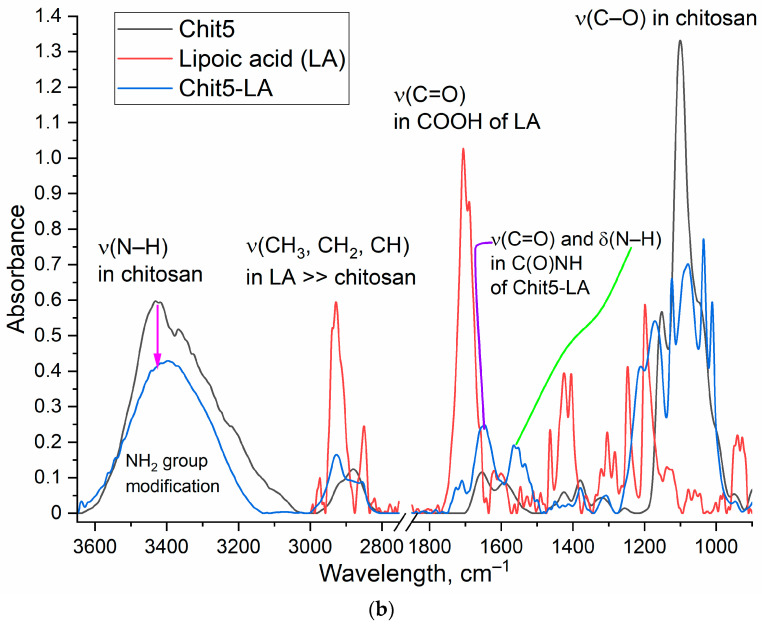
(**a**) The scheme of synthesis grafted chitosan with lipoic acid (Chit5-LA) with oleic acid (Chit5-OA). (**b**) FTIR spectra of Chit5, LA, and its conjugate Chit5-LA. T = 22 °C.

The synthesis of chitosan grafted with fatty acids was carried out using the carbodiimide approach described earlier [45,47]. The Chit5 and lipoic acid (LA) or oleic acid (OA) (Figure 3) chemical conjugation was confirmed by a significant decrease in intensity of the absorption band of carboxylic acid group (1730–1700 cm^−1^) of lipoic acid, and the appearance of characteristic peaks of ν(C=O) at 1630 cm^−1^ and δ(N–H) at 1560 cm^−1^ oscillation in amide bond between chitosan and acid residues. Conjugate formation is also confirmed by a decrease in the intensity of ν(N–H) at 3500–3300 cm^−1^ in NH_2_ groups of chitosan, since they are modified into amide. Grafted chitosan is characterized by three peaks of characteristic oscillation bands ν(C–H) in fatty acid residues at 3000–2850 cm^−1^. Interestingly, the structure of the C-O-C bond oscillation band (1200–1000 cm^−1^) in chitosan changes from two-component to multicomponent after modification with lipoic acid. This occurs due to the formation of micelles and various variants of the microenvironment of glucosamine fragments of chitosan.

^1^H NMR spectra of polymers with S-H or S-S bonds loaded with R6G are presented in Figure S2. Chit5-LA was studied as a self-assembled polymer with S-S bonds between chains or non-stitched S-H bonds. As a control without S-H and S-S bonds, Chit5-OA was used. Chemical shifts (δ, ppm) for Chit5 were observed: 4.22 (H1), 3.23 (H2), 3.79, 3.96 (H3, H4, H5, H6, H6′), and 2.11 (NH–C(=O)–CH_3_). ^1^H NMR spectra of Chit5-LA contain signals of chitosan indicated above, increased signals at 2.0–2.3 ppm and 1.25 ppm that were assigned to N-alkyl groups of LA, and signals of 3.64 ppm (C–H near the dithiolane fragment) and 2.3 ppm (β–H with relation to the carboxyl group) assigned to LA in polymer [45]. Upon thiol-disulfide exchange reaction, lipoic acid residues form intermolecular S-S inside the micelles (it was shown using NMR spectroscopy—Figure S2) accompanied by the particles compactization (the particle size decreases from 300–350 nm to 230–280 nm—Table 2, Figure S3), indicating increased thermodynamic stability (this is a consequence of the decline of the critical micelles concentration). The physicochemical properties of chitosan-based polymers and micelles formed from it are presented in Table 2. An increase in the zeta potential, when comparing Chit5-OA with Chit5-LA, indicates a change in the structure of the micelle and its greater homogeneity (acid residues look inward into the core). S-S crosslinking promotes the sealing of micelles and a decrease in the zeta potential.

#### 3.4.2. Polymeric Micelles Formation and S-S Stitching Detection Using MUTMAC–R6G Probe

**Figure 4:**
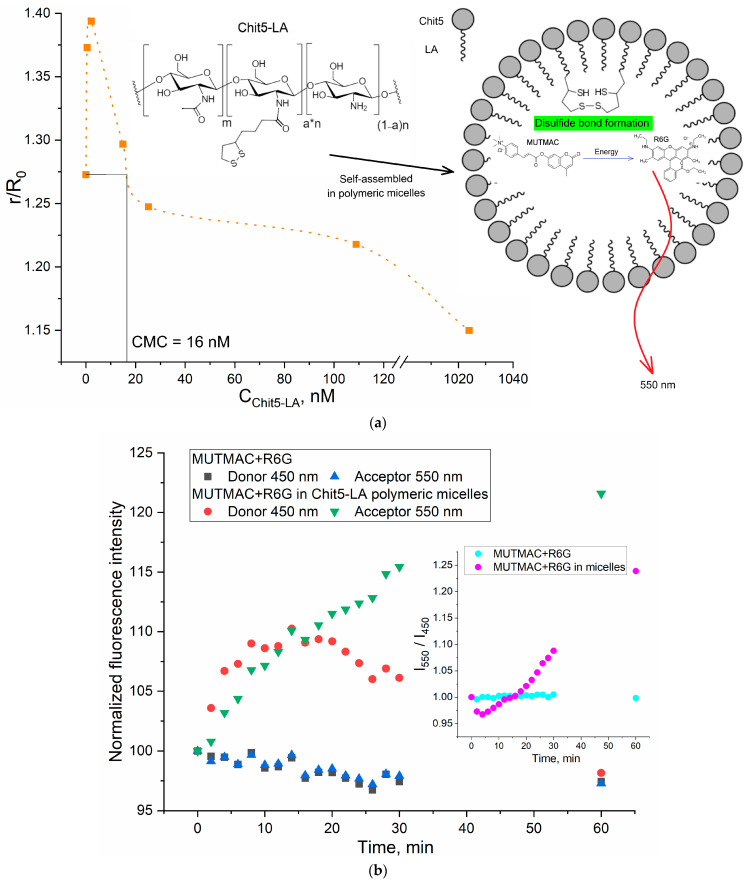

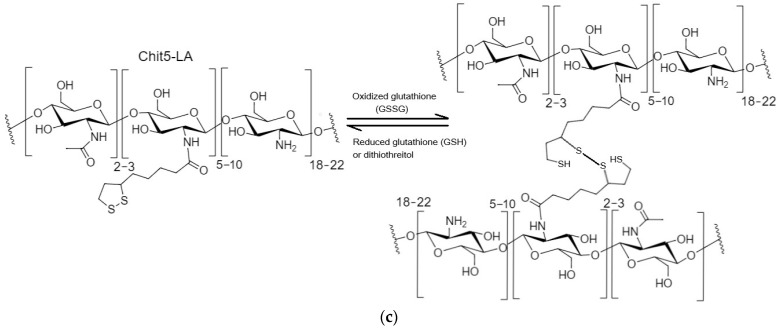
(**a**) The dependence of r/R_0_ (MUTMAC–R6G, 1 µM/1 µM) on the Chit5-LA self-assembled molecules concentration. r is the distance between donor and acceptor and R_0_ is Förster radius. The excitation wavelength is 360 nm. PBS buffer solution (0.01 M, pH 7.4). (**b**) Kinetic fluorescence curves of FRET probe components in buffer solution and during the formation of S-S bonds between crosslinking molecules of Chit5-LA. T = 37 °C. (**c**) The scheme for the formation and reducing of a disulfide bond.

To show the versatility of the FRET approach to determine CMC, in addition to surfactant micelles, we studied polymeric micelles based on the chitosan (5 kDa) grafted with lipoic or oleic acid (Chit5-LA, Chit5-OA). Figure 4a shows the distance between the fluorophores pair (donor–acceptor) plotted as a function of the grafted chitosan molecules concentration. The CMC determined for Chit5-LA is 16 nM (Table 2), which is in good agreement with the pyrene-probe data described earlier for similar chitosan-based micellar systems [45].

The developed FRET-based approach is further applied to study the mechanism and the kinetics of polymeric micelles formation. Of particular interest is the aspect of the formation of the polymeric micelles (as a smart drug delivery system), where the kinetics data of formation/destruction of S-S bonds are of great importance. This can be considered as the basis for creating stimulus-sensitive drug delivery systems to tumor cells, where drug molecules will be selectively released due to the higher glutathione level in cancer cells [45,47]. The visualization of the effective penetration of R6G into A549 cancer cells in the micellar form compared with a free cytostatic is shown in Figure S4.

Therefore, based on chitosan-lipoic acid conjugates (Figure 4a–c), we studied the kinetics of the polymeric micelles formation stabilized by covalent S-S bonds (the formation of disulfide bonds was confirmed by NMR spectroscopy (Figure S2)). Such micelles compactization results in strengthening of MUTMAC->R6G FRET (Figure 4b). An appropriate analytical signal here is the I_550_/I_450_ index (acceptor fluorescence/donor fluorescence)—the effectiveness of the FRET effect. There are no changes in the FRET status in the buffer solution. The formation of micelles was accompanied by the formation of a hydrophobic core and the compaction of (CH_2_)_n_ tails, while hydrophilic NH_2_ and OH groups are exposed out in the water. During the micelles’ formation, fluorescence increases, which indicates the inclusion of FRET probes in the hydrophobic areas of the micelle. On the other hand, the kinetic curve of the FRET signal (I550/I450 index) exhibits a minimum at approximately 3–5 min (Figure 4b, insert), which corresponds to an increase in FRET efficiency (in the micelles). During the formation of hydrophobic sites in the micellar particle, the drug (fluorophore) is loaded for about 5–10 min. After this point, we observe a subsequent linear increase in FRET signal up to ~1 h, which is due to the inclusion of FRET probes within the micelle cores and the continued process of micelle compactification during the formation of disulfide bonds. The crosslinking of polymeric chains in micelles (Figure 4c) causes compactization of structure; the micelle is thickened and the loading degree of FRET probes into hydrophobic core increases.

The FRET probes loading capacity was estimated to be equal to 75–80% for nonstitched polymeric micelles and 87% for S-S stitched. The crosslinking of polymer chains in micelles cause compactization of structure; the micelle is thickened and the degree of loading of FRET probes into hydrophobic venom increases. At the same time, the zeta potential of the micellar system decreases (Table 2). The dense structure of micelles is maintained at pH > 7 (typical for liquid media in body), while protonation of chitosan amino groups occurs in a weakly acidic medium, and loosening of micelles occurs with an increase in the rate of the drug release [45].

The inclusion of the studied fluorophores in polymeric micelles was demonstrated by flow cytometry (Figure S5) as a control technique, by the appearance of R6G-positive submicron micellar particles (R6G loading capacity was about 85%). The presence of fluorescent particles proves the predominant inclusion of rhodamine in the micellar system. Moreover, in the case of micelles containing covalent S-S bonds, the degree of inclusion of the fluorophore (according to the quenching of the fluorescence) is higher in comparison with nonstitched loose micelles.

**Figure 5:**
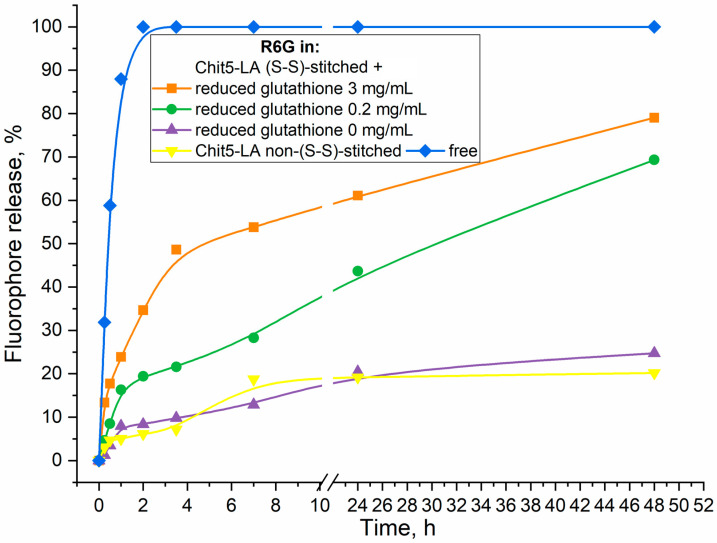
Release curves of rhodamine 6G (R6G) from Chit5-LA-based micelles not crosslinked with disulfide bonds and crosslinked with disulfide bonds in the presence of a reducing agent (glutathione). T = 37 °C. 0.01 M PBS (pH 7.4).

Polymeric micelles with disulfide bonds formation are investigated here as a perspective stimuli-sensitive drug delivery system to tumors. Reduced glutathione (GSH) is the most important antioxidant in cells [56,57], and was found in all cell compartments in millimolar concentrations (1–10 mM). Chitosan-based polymeric micelles use this feature of cancer cells: GSH as a trigger causes accelerated release of cytostatic [44]. Evidence of the formation of S-S bonds and the possibility of their destruction by a reducing agent (glutathione excess—tumor microenvironment model) are shown in Figure 5. An increase in the concentration of glutathione is reflected in a sharp (up to 5–10 times) increase in the rate of fluorophore release from Chit5-LA micelles due to the destruction of disulfide bonds. In other words, the release of fluorophore is characterized as glutathione-dependent: with an increase in the concentration of the thiol-disulfide exchange catalyst, disulfide bonds in micelles are reduced and the micelle structure is loosened with the simultaneous release of rhodamine 6G.

The release of the drug from the micelles is prolonged (Figure 5). At the same time, S-S crosslinked micelles, due to their dense structure, in the absence of GSH, release no more than 20% of the loaded drug. In the presence of GSH, the release rate becomes almost constant, while the full release of the drug is achieved in 3–4 days. The plateau (1) in the case of the presence of GSH is due to the limiting stage of S-S reduction and loosening of the micelle structure (the rate of release becomes constant and is approximately equal to 20–30% per day), and (2) in the absence of GSH, the inability to release the drug from a durable micelle (release was stopped at 15–20%). The plateau can be explained by the fact that the fluorophore molecules are released from covalent micelles only from the surface layers, while the inner parts remain tightly bound for a long period of time. This means that 20% of the drug is non-firmly bound, while 80% is deeply located and firmly bound to the micelle. At the same time, the drug is almost completely released under the action of specific stimuli (the microenvironment of tumor cells), which is a key advantage of the polymeric stimulus-sensitive micelle system.

Thus, we present two pairs of FRET probes (a more suitable MUTMAC–R6G pair was used for polymer micelles) that allowed us to monitor the formation of micellar structures from amphiphilic molecules or the kinetics of polymers self-assembling in real time.

### 3.5. A Comparison of the Proposed FRET Probe Technique with Other Techniques Described in the Literature to Study the Properties of Micelles

Table 3 compares the informativity of different methods used to study micelles characteristics, and evaluates the expressiveness and versatility of each approach. The advantages of the FRET technique are high sensitivity and the possibility to determine the distance between the fluorophores; thus, the mechanism of the micelles formation, CMC and CPMC values, and the size of the micelles can be estimated. At the same time, the method is fast and reproducible, since the FRET signal is rather specific.

## 4. Conclusions

In this work, we proposed the FRET probe technique (R6G with FITC or methylumbelliferone derivative, MUTMAC) as an indicator of micelle formation from surfactants or from chitosan grafted with fatty acid as promising drug carrier with stimuli-sensitivity to tumor microenvironments (pH is about 5.5–6.5 with increased concentrations of glutathione). In relation to surfactants (anionic SDS, cationic Zephirol, and nonionic Triton X-100), the FRET probe technique provides valuable information about the distance of the donor and acceptor fluorophores r/R_0_ (where R_0_ is the Förster distance and is about 50–60 Å), which was used to study the mechanism of micelle formation and to determine the aggregate state of the system (individual molecules/pre-micelles/micelles) and the CMC parameters. Chitosan grafted with oleic and lipoic acid was synthesized using the carbodiimide approach, followed by characterization by FTIR and NMR spectroscopy: grafting degree is about 20%, average molecular weight per one structure unit is about 6–7 kDa for Chit5-OA and non-stitched Chit5-LA, but for Chit-LA S-S stitched, molecular weight is about 45 kDa (6–8 fragments). Chitosan conjugates self-assemble into positively charged (+5–20 mV) polymeric micelles when concentration is higher than 10–20 nM. Micelles formation and functional properties, such as fluorophore loading degree, were studied using the FRET technique and were also controlled by flow cytometry and atomic force microscopy. Reductant-treated conjugate Chit5-LA, due to S-S crosslinks formation between polymer chains via lipoic acid residues, is accompanied by particles’ compactization (the particle size decreases from 300–350 nm to 230–280 nm). One of the key aspects of the work is the effect of the formation and destruction of S-S bonds between polymer chains in micelles on FRET efficiency, which is important in the development of stimulus-sensitive drug delivery systems for antitumor therapy. The release of R6G (model cytostatic and fluorophore) is characterized as glutathione-dependent: with an increase in the concentration of the thiol-disulfide exchange catalyst, S-S bonds in micelles are reduced to S-H and the micelle structure is loosened with the simultaneous release of rhodamine 6G. Thus, we presented the original technique of FRET probes in relation to the study of micelle formation processes of various amphiphilic molecules, and, most importantly, demonstrated the applicability of FRET probes to study the characteristics of micellar drug delivery systems with the function of active tumor targeting.

## Figures and Tables

**Table 2:** Physicochemical properties of chitosan-based polymeric micelles. T = 22 °C.

Micelle *	Grafting Degree, %	M_w_ of One Polymeric Unit, kDa	CMC, nM	Hydrodynamic Diameter **, nm	Zeta Potential, mV
Chit5-OA	18 ± 2	6.7 ± 0.8	8 ± 2	300–450	+5 ± 1
Chit5-LA nonstitched	24 ± 3	6.4 ± 0.3	50 ± 10	300–350	+20 ± 3
Chit5-LA S-S stitched		45 ± 6 (is about 7 residues of Chit5-branches)	16 ± 2	230–280	+15 ± 2

* Chit5—chitosan 5 kDa. OA—oleic acid without SH and S-S groups. LA—lipoic acid with SH or/and S-S groups. ** Determined by nanoparticle tracking analysis (NTA).

**Table 3:** A comparison of the methods used to investigate the micelles’ properties. “+” means that the method provides relevant information about the parameter. “++” means that the method provides comprehensive information about the parameter. “±” means that the method provides indirect information about the parameter. “−” means that the method does not provide information about the parameter or the data does not follow directly.

Method	CMC Determination	CPMC Determination	Aggregation Number Determination	Size Determination	Robustness	Applicability to Different Types of Micelles	Expressiveness
Conductometry [39,58,59]	±	−	−	−	+	±	+
Surface tension [51,60,61]	+	−	−	−	±	−	+
Densitometry [59]	±	−	−	−	±	−	+
NMR spectrometry [62]	+	±	±	−	+	±	−
UV/VIS spectroscopy [63]	+	−	−	−	+	±	+
Fluorometric methods (including pyrene probe) [32,61,64,65,66]	++	+	±	±	+	+	+
Atomic force and electron microscopy [67]	±	−	−	++	±	+	−
FRET probes	+	+	±	±	+	++	+

## Data Availability

The data presented in this study are available in the main text.

## Supplementary Materials

The following supporting information can be downloaded at: https://www.mdpi.com/article/10.3390/polym16060739/s1, Figure S1. The dependence of the fluorescence emission maximum intensity on surfactant concentration: (a) MUTMAC, (b) FITC, (c) R6G. Fluorescence emission spectra of (d) FITC, (e) R6G in free form and in micellar form. Graphs were used to determine the degree of incorporation of fluorophore into micelles. Figure S2. ^1^H NMR of polymeric micellar systems with S-H or S-S bonds with R6G. Chit5-LA was studied as self-assembled polymer. As a control without S-H bonds, Chit5-OA was used. D_2_O. T = 25 °C. Figure S3. (a) AFM image of Chit5-LA particles and (b) the corresponding section along the blue line in height, respectively. Figure S4. Fluorescence images of A549 after 60 min incubation with Rhodamine 6G 1 μg/mL free or Rhodamine 6G in micelles (S-S stitched). R6G red, DAPI blue, and FITC-labeled micelles channels and merge are shown. R6G/micelle 1:1 *w*/*w*. The scale segment is 100 µm. Figure S5. Flow cytometry assay of R6G-loaded micelles.

## Abbreviations

Chit—chitosan; CMC—critical micelle concentration; CPMC—critical pre-micelle concentration; FITC—fluorescein isothiocyanate; FRET—Förster resonance energy transfer; LA—lipoic acid; MUmb—4-methylumbelliferone or methylumbelliferyl; MUTMAC—4-methylumbelliferyl p-trimethylammoniocinnamate chloride; R6G—rhodamine 6G; SDS—sodium dodecyl sulfate.
