# Supplementary material for: Specific FRET Probes Sensitive to Chitosan-Based Polymeric Micelles Formation, Drug-Loading, and Fine Structural Features
Source: Polymers (Basel). 2024 Mar 8;16(6):739. doi: 10.3390/polym16060739 (PMC10975940; doi:10.3390/polym16060739)

Specific FRET probes sensitive to chitosan-based polymeric micelles formation, drug-loading and fine structural features

Igor D. Zlotnikov, Ivan V. Savchenko and Elena V. Kudryashova *

Faculty of Chemistry, Lomonosov Moscow State University, Leninskie Gory, 1/3, 119991 Moscow, Russia
* Correspondence: helenakoudriachova@yandex.ru

Figure S1. The dependence of the fluorescence emission maximum intensity on surfactant concentration: **(a)** MUTMAC, **(b)** FITC, **(c)** R6G. Fluorescence emission spectra of **(d)** FITC, **(e)** R6G in free form and in micellar form. Graphs were used to determine the degree of incorporation of fluorophore into micelles.

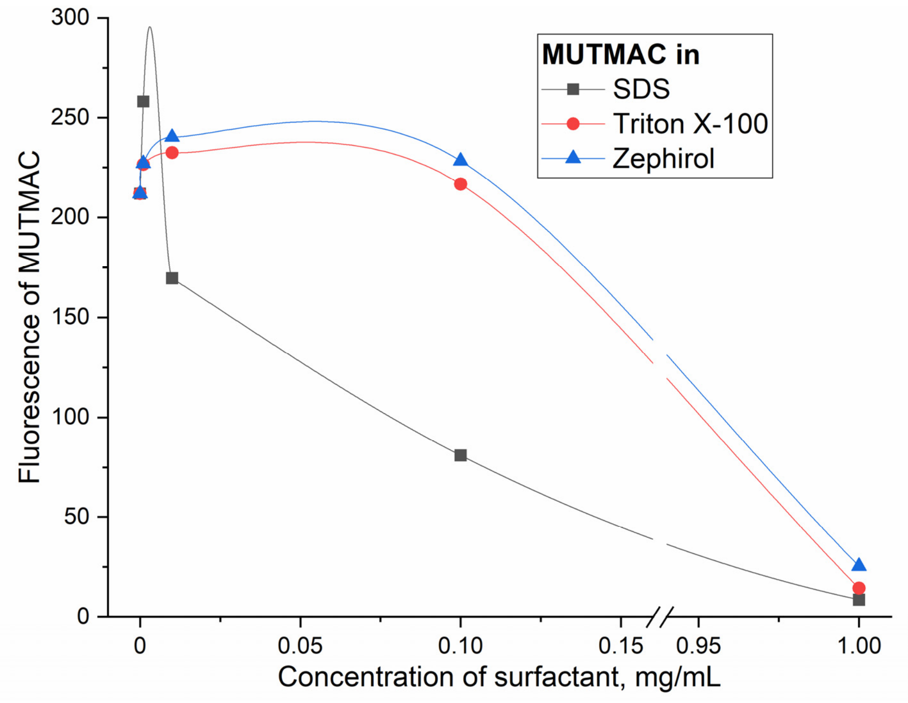

(a)

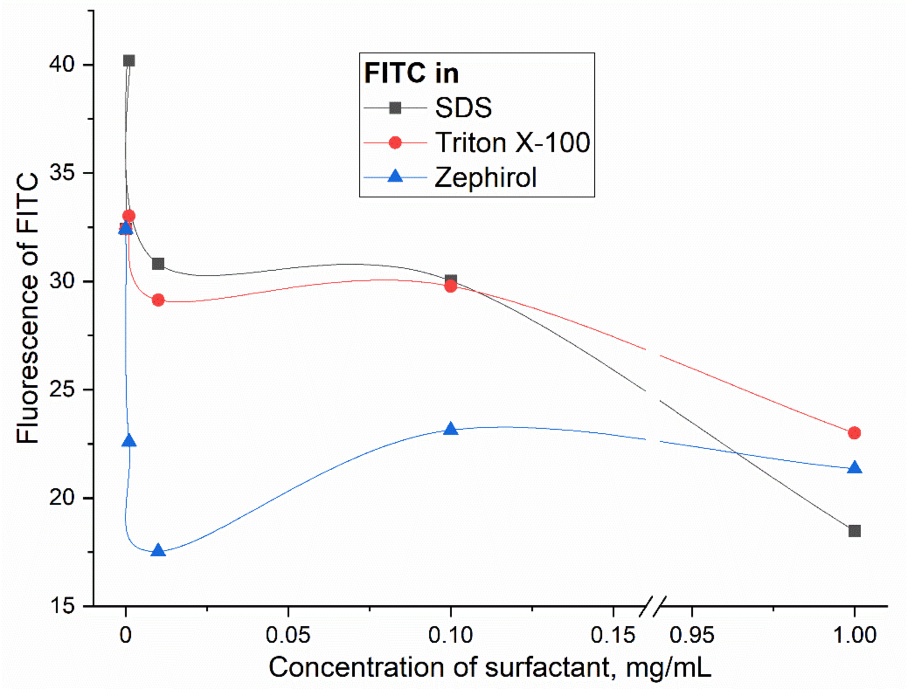

(b)

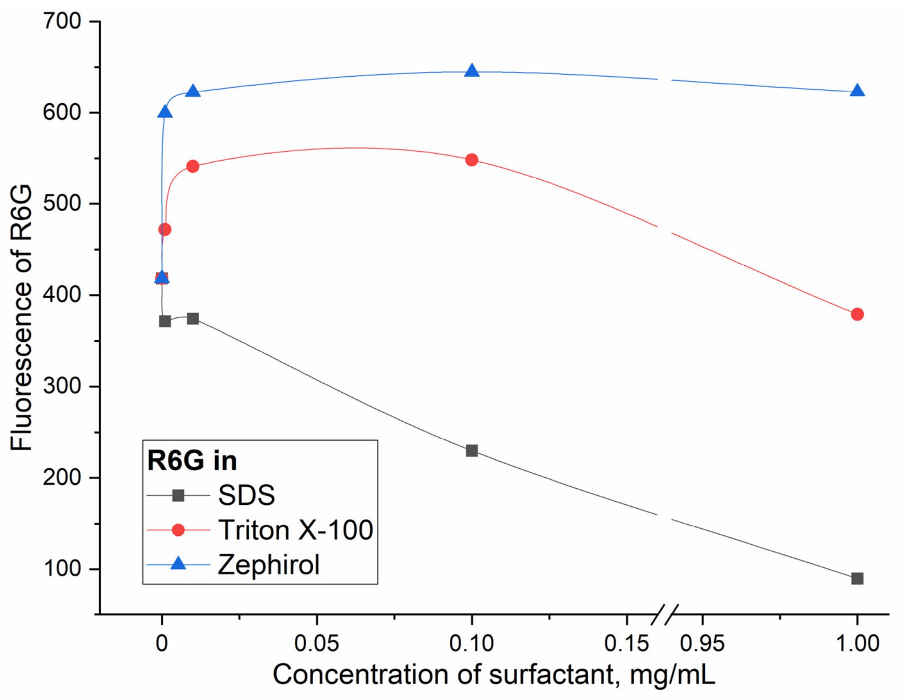

700
600
500
400
300
200
100
Fluorescence of R6G
R6G in
SDS
Triton X-100
Zephirol
0
0.05
0.10
0.15
0.95
1.00
Concentration of surfactant, mg/mL

(c)

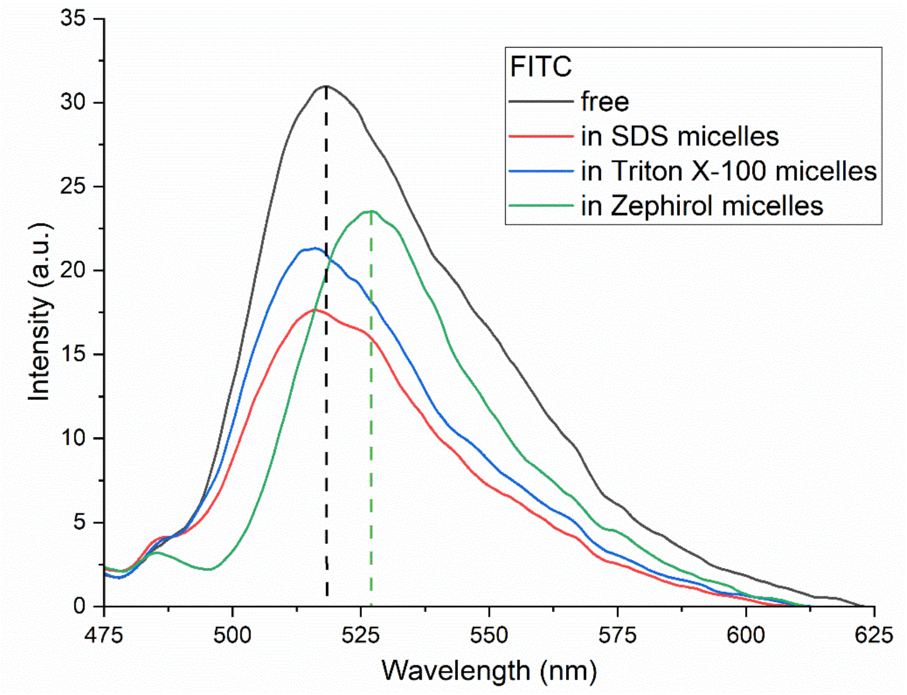

35
30
25
20
15
10
5
0
Intensity (a.u.)
FITC
free
in SDS micelles
in Triton X-100 micelles
in Zephirol micelles
475
500
525
550
575
600
625
Wavelength (nm)

(d)

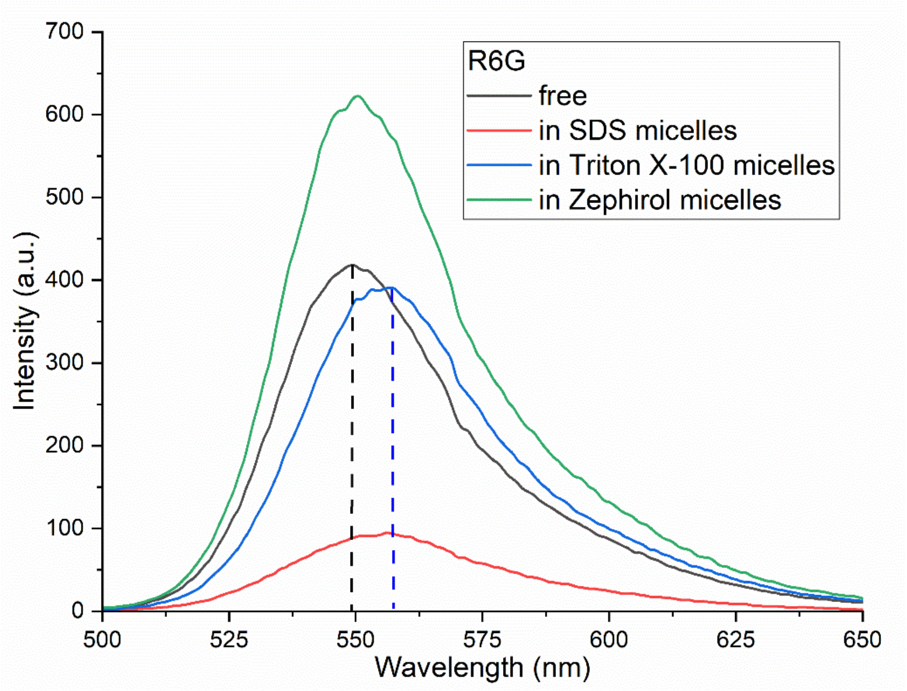
R6G
free
in SDS micelles
in Triton X-100 micelles
in Zephirol micelles
Intensity (a.u.)
Wavelength (nm)
0
100
200
300
400
500
600
700
500
525
550
575
600
625
650

(e)

Figure S2. ^{1}H NMR of polymeric micellar systems with S-H or S-S bonds with R6G. Chit5-LA was studied as self-assembled polymer. As a control without S-H bonds Chit5-OA was used. D_2O. T = 25 °C.

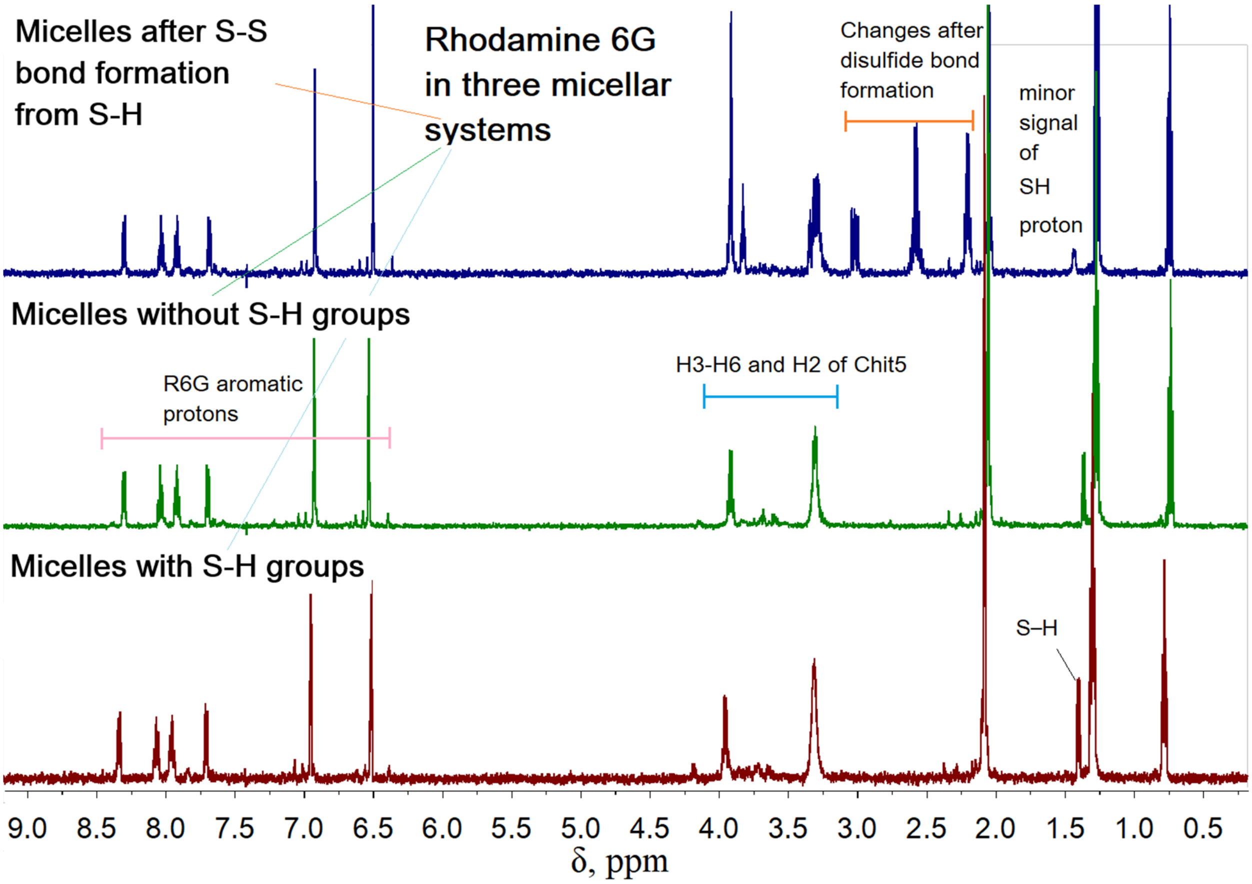

Figure S3. (a) AFM image of Chit5-LA particles and (b) the corresponding section along the blue line in height, respectively.

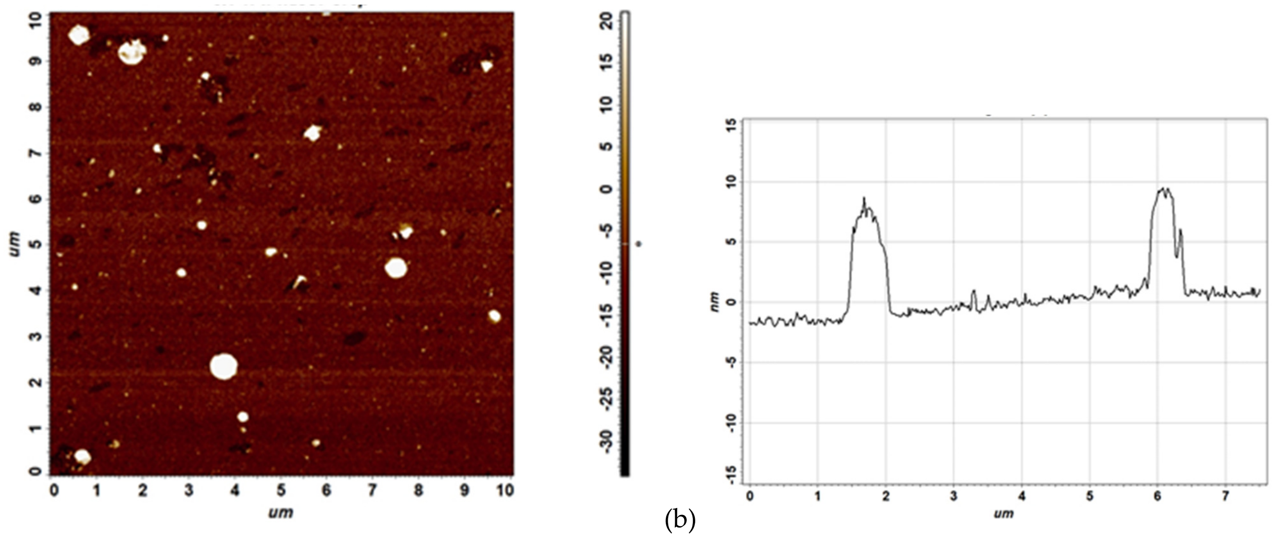

(a) (b)

Figure S4. Fluorescence images of A549 after 60 min incubation with Rhodamine 6G 1 μg/mL free or Rhodamine 6G in micelles (S–S stitched). R6G red, DAPI blue, and FITC-labelled micelles channels and merge are shown. R6G/micelle 1:1 w/w. The scale segment is 100 μm.

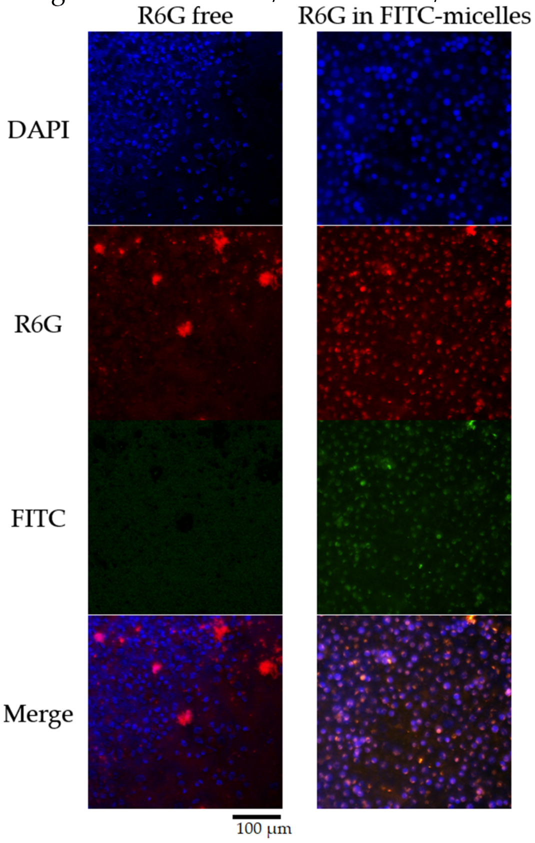

Figure S5. Flow cytometry assay of R6G-loaded micelles.

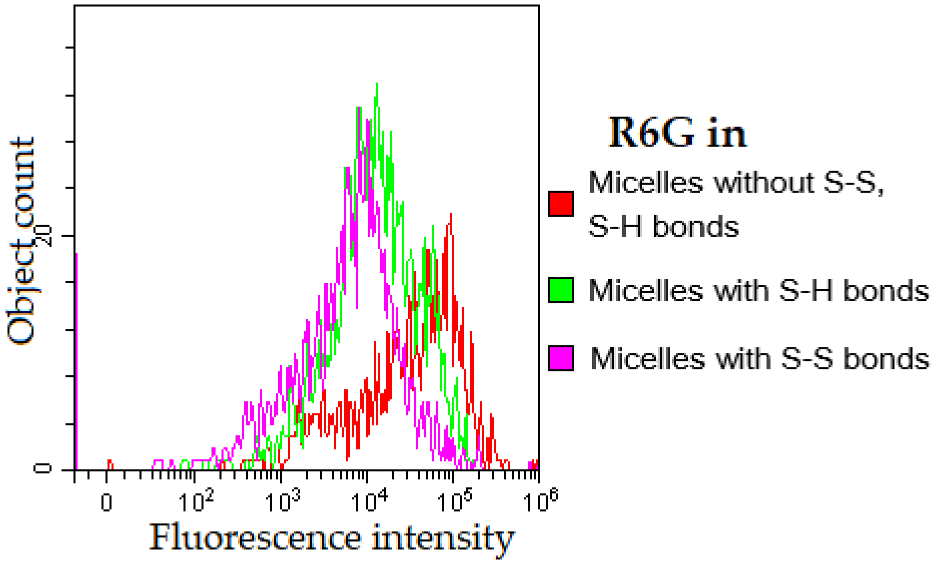

Supplement: Supplementary file 1 [file polymers-16-00739-s001.zip › polymers-2855885-supplementary.pdf]
